# *Akkermansia muciniphila* as a Potential Guardian against Oral Health Diseases: A Narrative Review

**DOI:** 10.3390/nu16183075

**Published:** 2024-09-12

**Authors:** Molly H. Anderson, Karima Ait-Aissa, Amal M. Sahyoun, Ammaar H. Abidi, Modar Kassan

**Affiliations:** College of Dental Medicine, Lincoln Memorial University, LMU Tower, 1705 St. Mary Street, Knoxville, TN 37917, USA; molly.anderson@lmunet.edu (M.H.A.); karima.aitaissa@lmunet.edu (K.A.-A.); amal.sahyoun@lmunet.edu (A.M.S.)

**Keywords:** *Akkermansia muciniphila*, short chain fatty acids, dysbiosis, inflammation, periodontal disease, obesity, diabetes, hypertension, oral health, systemic diseases, probiotics, prebiotics, diet

## Abstract

The oral microbiome is a diverse ecosystem containing a community of symbiotic, commensal, and pathogenic microorganisms. One key microorganism linked to periodontal disease (PD) is *Porphyromonas gingivalis* (*P. gingivalis*), a Gram-negative anaerobic bacterium known to have several virulence factors that trigger inflammation and immune evasion. On the other hand, *Akkermansia muciniphila* (*A. muciniphila*), a symbiotic bacterium, has been recently shown to play an important role in mitigating inflammation and reducing periodontal damage. In vivo and in vitro studies have shown that *A. muciniphila* decreases inflammatory mediators and improves immune responses, suggesting its role in mitigating PD and related inflammatory systemic conditions such as diabetes, hypertension, and obesity. This review discusses the anti-inflammatory effects of *A. muciniphila*, its impact on periodontal health, and its potential role in managing systemic diseases. The overall aim is to elucidate how this bacterium might help reduce inflammation, improve oral health, and influence broader health outcomes.

## 1. Oral Health and Inflammation

The oral cavity has an intricate and diverse population of microorganisms that play a crucial role in oral health and disease. This complex and dynamic community of microorganisms together are known as the host’s oral microbiome [1]. Oral health plays a pivotal role in maintaining systemic health, with the mouth serving as a gateway to the rest of the body. Healthy oral parameters such as intact teeth, healthy gums, and a balanced oral microbiota are essential for preventing not only dental diseases but also systemic conditions. Beneficial bacteria, such as *Streptococcus salivarius* and *Lactobacillus* spp., help maintain a neutral pH, inhibit the growth of pathogens, and support the immune response. However, when oral hygiene is neglected or compromised, there can be a dysbiotic shift in the oral microbiota, where harmful bacteria such as *Porphyromonas gingivalis* and *Fusobacterium nucleatum* begin to dominate [2]. A balanced oral microbiota is essential for preventing inflammation and conditions like gingivitis and periodontitis [3]. The equilibrium between beneficial (symbiotic) and harmful (pathogenic) bacteria is delicate; when disrupted (dysbiosis), it can lead to an overgrowth of pathogenic bacteria, resulting in inflammation and subsequent oral diseases [4].

Recent research signifies the importance of maintaining a balanced oral microbiota, which aids in preventing uncontrolled inflammation [5]. It is evident by many scientific studies that maintaining the balance of the oral microbiome is key to oral health, as it helps regulate immune responses and inhibit the growth of harmful bacteria [6]. A well-balanced oral microbiota not only protects against local inflammation but also has broader implications for systemic health [7]. The connection between oral health and systemic health is increasingly recognized, as chronic oral infections like periodontitis can lead to a persistent inflammatory state in the body. Inflammation in the oral cavity has been linked to various systemic conditions, including cardiovascular diseases, diabetes, and respiratory infections [8]. This inflammation, driven by the pathogenic bacteria and their byproducts, can contribute to the progression of systemic diseases. For example, *Porphyromonas gingivalis*, a key player in periodontitis, has been implicated in the pathogenesis of atherosclerosis by invading endothelial cells and promoting plaque formation in arteries [9]. Given the interconnectedness of the body’s systems, it’s essential to understand how gut microbiota influences oral health. Therefore, maintaining good oral hygiene is crucial not only for preventing oral diseases but also for safeguarding overall health by controlling oral inflammation and maintaining a balanced oral microbiota, individuals can reduce their risk of developing serious systemic health issues.

**Figure 1:**
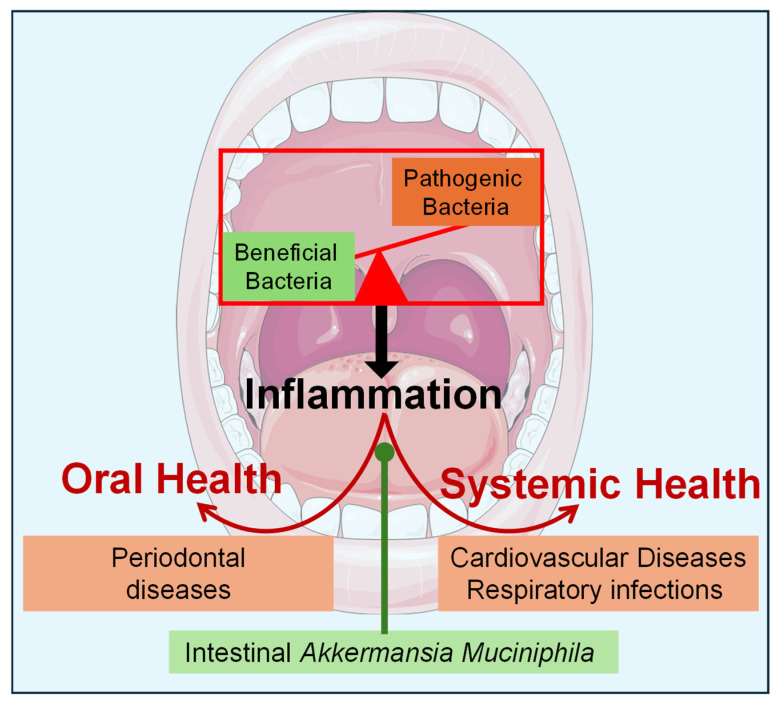
Oral Bacterial Imbalance, Inflammation, and the Protective Role of *Akkermansia muciniphila*. Imbalance in oral bacteria leads to inflammation, which contributes to periodontal diseases and can impact systemic health. *Akkermansia muciniphila*, a gut bacterium with anti-inflammatory properties, can help alleviate these conditions by reducing inflammation and promoting oral and overall health. Red arrows indicate the potentiation or worsening of diseases due to inflammation, while green arrows signify the inhibition or alleviation of diseases by decreasing inflammation.

*A. muciniphila*, a beneficial commensal bacterium predominantly found in the gut, has recently garnered attention for its potential role in oral health [10]. Known for its anti-inflammatory properties, *A. muciniphila* has been shown to regulate immune responses and reduce inflammation, making it a promising candidate for the maintenance of oral health as well [11]. Studies indicate that *A. muciniphila* can decrease the production of pro-inflammatory cytokines and enhance the anti-inflammatory response, suggesting its potential to mitigate oral inflammation and improve periodontal health [10] (Figure 1).

This review article seeks to investigate the role of the oral microbiota, particularly *A. muciniphila*, in reducing inflammation, enhancing oral health, and impacting overall health outcomes. The review examines the latest research findings and discusses the mechanisms through which *A. muciniphila* regulates inflammation and the potential therapeutic strategies for maintaining the microbial communities in balance. The current review aims to offer insights into effective strategies for the prevention and management of oral and systemic inflammatory diseases.

## 2. *Akkermansia muciniphila* and Inflammation

The relationship between the human microbiome and periodontal diseases (PD) is intricate and multifactorial. The gut microbiome and the oral microbiome play crucial roles in the development and progression of PD. This primarily occurs due to dysbiosis—an imbalance in the microbial communities—along with the presence of pathogenic bacteria that provoke an inflammatory response, eventually leading to PD. The human gut microbiome orchestrates a delicate equilibrium between vitality and illness, embodying the fine line that separates health from disease. The gastrointestinal tract (GI) microbiota has a profound impact on human physiology, influencing both normal functioning and pathological states [12]. Several microbial species have gained attention for their role in regulating the GI microbiota [13]. *A. muciniphila* is known to improve immune response and metabolic functions. As a strict anaerobe, it relies exclusively on mucin as its primary source of carbon and nitrogen elements. Mucins are an integral component of the protective mucus layer that lines the intestinal epithelium. It was once thought that mucin degradation could be detrimental to the host. However, this process is crucial, given the abundant production of mucins in the intestinal tract. Yet, it’s important to recognize that excessive mucin production can also pose problems, highlighting the need for balanced regulation within the gut. Mucin production serves as a crucial energy source primarily for colonic bacteria, supporting the host’s microbial ecosystem [14]. By degrading mucin, *A. muciniphila* plays a critical role in maintaining the integrity of the mucus barrier and preventing the infiltration of pathogens or inflammatory markers into the gut tissue [13].

Inflammation is a significant factor in the development of various systemic conditions, and one way the gut microbiota helps regulate inflammation is through the production of short-chain fatty acids (SCFAs). SCFAs are a specific group of fatty acids synthesized by the intestinal microbiota following the fermentation of nondigestible polysaccharides. The major SCFAs produced by this process include acetate, propionate, and butyrate. The proximal colon contains the highest concentrations of SCFAs, which are either utilized locally by enterocytes or transported across the intestinal epithelium into the bloodstream [15]. *A. muciniphila* produces SCFAs, primarily acetate, and propionate, through specialized breakdown of heavily O-glucosylated mucins [16]. SCFAs exhibit anti-inflammatory properties and contribute to the preservation of intestinal barrier integrity, resulting in reduced gut and systemic inflammation [14]. As a result, the presence of *A. muciniphila* enhances SCFA production, which in turn helps reduce inflammation and lowers the risk of associated diseases.

**Figure 2:**
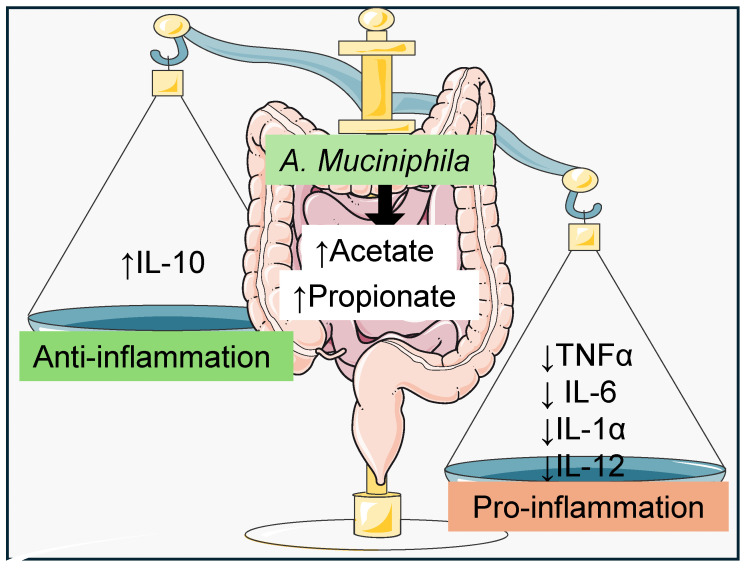
*Akkermansia. muciniphila* and inflammation. *A. muciniphila* by increasing acetate and propionate levels reduces pro-inflammatory cytokines (TNF-α, IL-6, IL-1α, IL-12) while simultaneously increasing the anti-inflammatory cytokine IL-10. These findings highlight the crucial role of *A. muciniphila* in mitigating inflammation and highlight its potential as a therapeutic agent. Symbol ↑ indicates upregulation/increase and symbol ↓ indicates downregulation/decrease.

The interplay between *A. muciniphila* and interleukin-10 (IL-10), an anti-inflammatory cytokine, is crucial for elucidating their roles in modulating inflammatory processes and preserving gut health. IL-10 plays a key role in restraining inflammatory responses, thereby safeguarding the host from potential tissue damage [17]. Previous studies have explored the connection between *A. muciniphila* and host metabolic inflammation, demonstrating that *A. muciniphila* has anti-inflammatory properties within the intestinal mucosal barrier. Levels of *A. muciniphila* are notably lower in individuals with inflammatory bowel diseases, such as Crohn’s disease and ulcerative colitis, which adversely affects the organism’s ability to protect against inflammatory intestinal injury [18]. In an in vivo study using a Dextran sodium sulfate (DSS)-induced colitis mouse model, the anti-inflammatory effects of *A. muciniphila* were demonstrated. Daily oral administration of *A. muciniphila* for 14 days significantly improved colitis by reducing pro-inflammatory cytokines (TNF-α, IL-6, IL-1α, IL-12A) in both serum and colon tissue, while also increasing IL-10 levels [19]. Collectively, these findings expose the pivotal role of *A. muciniphila* in mitigating intestinal inflammation through modulation of pro-inflammatory cytokines and enhancement of IL-10 levels, highlighting its potential therapeutic implications (Figure 2).

## 3. *Akkermansia muciniphila* and Oral Health

The oral microbiome consists of diverse microorganisms, with some supporting oral health while others may contribute to PD. One of the predominant bacterial species implicated in periodontal pathology is *Porphyromonas gingivalis* (*P. gingivalis*) [20]. *P. gingivalis*, a Gram-negative anaerobic bacterium, elicits a host response through the action of virulence factors, encompassing both secretory and structural components. Notably, the fimbriae of *P. gingivalis* facilitate an augmentation of the inflammatory response while enabling evasion of host immune clearance mechanisms [9]. *P. gingivalis* lipopolysaccharide (LPS) is an important virulence factor that induces inflammation in the periodontal pocket [21]. LPS is a major component of the cell wall of Gram-negative bacteria, and it is well known for its toxicity. In response to this toxicity, host cells initiate inflammatory responses in the gingival tissue. This fosters an environment conducive to the progression of PD by providing optimal conditions for the proliferation of pathogenic microorganisms [22].

The presence of *A. muciniphila* has been shown to reduce pro-inflammatory cytokine production and decrease gastrointestinal inflammation, though research on its effects within the oral microbiome is limited [23]. A recent study explored the anti-inflammatory effects of *A. muciniphila* on PD and alveolar bone loss. Specifically, in vivo acute or chronic *P. gingivalis* infections treated with *A. muciniphila* showed significant reductions in bone tissue destruction in both lean and obese mice. Moreover, *A. muciniphila* supplementation enhanced macrophage M2 anti-inflammatory responses, evidenced by increased IL-10 and decreased IL-12 production [24]. Another study examined the effects of *A. muciniphila* on periodontal destruction in lean and obese mice with induced *P. gingivalis* infections. After three weeks of infection, *A. muciniphila* was administered orally or via gastric gavage for another three weeks. The results showed that oral administration of *A. muciniphila* significantly reduced periodontal destruction and inflammation, decreased plasma TNF-α levels, and increased IL-10 levels in both lean and obese mice [25].

**Figure 3:**
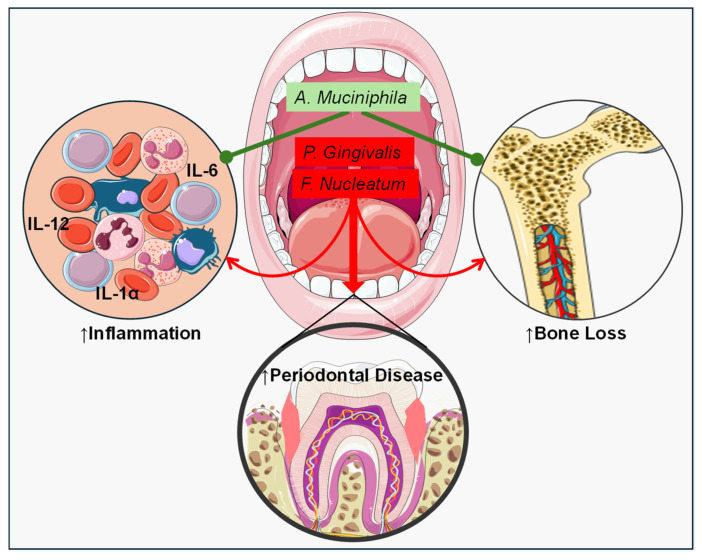
*A. muciniphila* and Oral Health-induced inflammation and bone loss. *Porphyromonas gingivalis* (*P. gingivalis*) and *Fusobacterium nucleatum* (*F. nucleatum*) have detrimental effects on periodontal diseases (PD) by triggering inflammatory responses and bone destruction. *Akkermansia muciniphila* has been shown to reduce inflammation, mitigate bone destruction, and enhance anti-inflammatory responses in models of PD. These findings highlight the potential of *A. muciniphila* as a therapeutic agent in the management of PD-associated inflammation and bone loss. Red arrows indicate the potentiation or worsening of diseases due to inflammation and bone loss, while green arrows signify the inhibition or alleviation of diseases by decreasing inflammation and bone destruction. Symbol ↑ indicates increase.

An additional research study investigated the impact of *A. muciniphila* on *Fusobacterium nucleatum* (*F. nucleatum*) and its potential to mitigate periodontitis. *F. nucleatum* is a Gram-negative, obligate anaerobic bacterium frequently associated with PD. Its presence is strongly correlated with periodontal inflammation, as it can independently cause significant inflammation and alveolar bone destruction [10]. In this study, gingival epithelial cells (GECs) were supplemented with *F. nucleatum* and *A. muciniphila*. Transcriptome sequencing and ELISA experiments demonstrated that *A. muciniphila* mitigated the inflammatory effects of *F. nucleatum* on GECs and reduced the secretion of inflammatory factors. When mice were inoculated with *F. nucleatum* and/or *A. muciniphila. F. nucleatum* alone led to increased alveolar bone resorption and a higher number of inflammatory cells. In contrast, mice inoculated with *A. muciniphila* showed reduced alveolar bone resorption and fewer inflammatory cells in the gingiva [10]. These findings demonstrate the promising potential of *A. muciniphila* in mitigating PD-associated inflammation and alveolar bone loss, offering avenues for further exploration in oral health research (Figure 3).

## 4. *Akkermansia muciniphila* and Other Systemic Diseases Related to Oral Health

There is evidence that supports a robust cause-and-effect relationship between PD and numerous systemic conditions, including hypertension and obesity [26]. The relationship between inflammation and PD is bidirectional: PD contributes to systemic inflammation, which, in turn, exacerbates the progression of PD. *A. muciniphila* is recognized for its ability to mitigate inflammation, consequently offering therapeutic benefits across various diseases, as evidenced in both murine models and human studies [18]. These findings highlight the potential therapeutic role of A. muciniphila in treating inflammation-related systemic conditions. The connection between PD and inflammation further emphasizes the complex relationship between oral and systemic health.

Obesity is recognized as one of the most serious public health challenges of the 21st century due to its profound negative impact on overall quality of life [27]. Obesity is a complex condition that serves as a major pathway to numerous chronic diseases. It is estimated to cause 3.4 million deaths globally and affects over 671 million people worldwide [28]. Recent studies have demonstrated that *A. muciniphila* improves various obesity parameters. An in vivo study observed that body weight gain, caloric intake, mesenteric fat weight, subcutaneous fat weight, epididymal fat weight, total fat, and energy efficiency significantly decreased in high fat diet (HFD)-fed mice following treatment with pasteurized *A. muciniphila* culture. Moreover, bacterial supplementation with *A. muciniphila* led to increased mRNA levels of Peptide YY (PYY) and a significant upregulation of Glucagon-like peptide-1 (GLP-1) gene expression, both of which are intestinal hormones with appetite-suppressing, anti-diabetic, and anti-obesity properties [29]. Interestingly, a clinical study has investigated the effects of *A. muciniphila* on humans through a randomized, double-blind, placebo-controlled pilot trial involving overweight/obese insulin-resistant volunteers. The study showed that daily oral supplementation with live or pasteurized *A. muciniphila* for three months was safe and well-tolerated. Compared to a placebo, *A. muciniphila* improved insulin sensitivity, reduced insulinemia, and lowered total cholesterol. Pasteurized *A. muciniphila* also led to slight decreases in body weight, fat mass, and hip circumference. Moreover, after three months, it reduced blood markers linked to liver dysfunction and inflammation, while leaving the overall gut microbiome unchanged. [30].

Studies suggest that *A. muciniphila* plays a role in decelerating the development and advancement of diabetes. Substrates like metformin, betaine, and tryptophan have been found to stimulate the growth of *A. muciniphila*. Metformin, a key anti-diabetic drug, not only reduces liver glucose production and improves insulin sensitivity but also promotes *A. muciniphila* proliferation, helping to lower the risk of type 2 diabetes (T2D) [14]. Numerous in vivo studies have illustrated the inverse correlation between *A. muciniphila* and T2D. High-fat diet (HFD)-fed mice displayed increased inflammation, hyperinsulinemia, and hyperglycemia, leading to T2D and a decrease in *A. muciniphila* levels. In contrast, mice without type 2 diabetes had higher levels of *A. muciniphila* [31,32].

Hypertension (HTN) remains a leading cause of mortality worldwide [33]. The intricate link between HTN and dysbiosis of the gut microbiome has been well-determined [34]. Gut microbiota dysbiosis can contribute to HTN through various pathways, including its influence on the immune system via SCFAs, modulation of the sympathetic nervous system, involvement in the trimethylamine-N-oxide (TMAO) pathway, and the promotion of chronic inflammation [35,36]. Interestingly, *A. muciniphila* has shown a potential role in regulating numerous metabolites, encompassing SCFAs, LPS, and TMAO [37,38].

**Figure 4:**
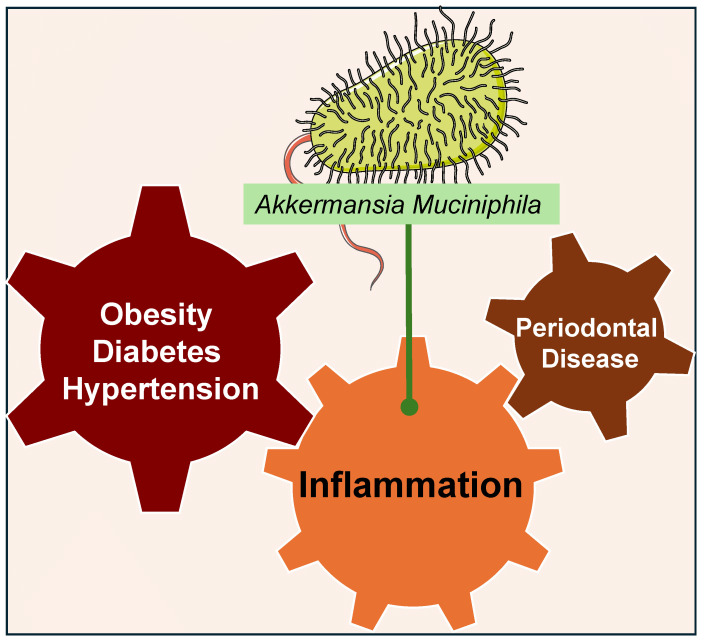
*Akkermansia muciniphila* and Other Systemic Diseases Related to Oral Health. *Akkermansia muciniphila* (*A. muciniphila*) has emerged as a notable player in managing systemic conditions linked to inflammation, including periodontal disease (PD), obesity, type 2 diabetes (T2D), and hypertension (HTN). *A. muciniphila*’s influence on inflammatory pathways and metabolic parameters highlights its potential as a therapeutic agent for chronic diseases and emphasizes the importance of the gut microbiome in overall health. Green arrows signify the inhibition or alleviation of diseases by decreasing inflammation.

These findings highlight *A. muciniphila*’s significant potential as a therapeutic agent for managing inflammation and related systemic conditions. Its capacity to influence inflammatory pathways and metabolic parameters presents promising opportunities for future research and clinical applications. The complex relationship between *A. muciniphila* and chronic diseases underscores the pivotal role of the human microbiome in maintaining overall health and addressing major public health challenges such as obesity, T2D, and HTN. Further exploration of these microbial interactions will be crucial in developing innovative treatments and enhancing health outcomes (Figure 4 and Table S1).

## 5. Clinical Implications

The growing research on *A. muciniphila* offers promising clinical implications, particularly in the realm of PD and its associated systemic conditions. By modulating the oral microbiota, *A. muciniphila* presents a novel therapeutic avenue to combat chronic inflammation and its detrimental effects on both oral and overall health.

The anti-inflammatory properties of *A. muciniphila* has significant potential for managing PD. Its ability to downregulate pro-inflammatory cytokines and enhance anti-inflammatory responses can help mitigate tissue destruction and bone loss caused by pathogens such as *P. gingivalis*. Clinical strategies incorporating *A. muciniphila* could lead to innovative treatments that reduce the prevalence and severity of PD.

PD is closely associated with systemic conditions including obesity, T2D, and HTN, reflecting the complex relationship between oral health and systemic health. *A. muciniphila*’s role in improving insulin sensitivity, reducing cholesterol levels, and modulating inflammatory markers underscores its potential as a therapeutic agent beyond oral health. By addressing the underlying inflammation, *A. muciniphila* could play a critical role in managing these systemic conditions, thereby improving patient outcomes.

The integration of *A. muciniphila* in probiotic and prebiotic formulations represents a promising clinical application that could help maintain a balanced oral microbiota, reducing inflammation and supporting overall health. Clinical trials focusing on the efficacy and safety of these formulations in diverse populations are essential to validate their therapeutic potential.

The variability in individual microbiomes suggests that personalized approaches to incorporating *A. muciniphila* could optimize therapeutic outcomes. Tailoring treatments based on a patient’s specific microbiota composition and inflammatory profile could enhance the effectiveness of interventions and minimize adverse effects.

Continued investigative efforts into the mechanisms by which *A. muciniphila* exerts its beneficial effects are crucial. Understanding its interactions with other microbial species and host immune responses will inform the development of targeted therapies. Longitudinal studies examining the impact of *A. muciniphila* supplementation on oral and systemic health over time will provide deeper insights into its clinical applications. While direct therapeutic use of *A. muciniphila* is currently limited due to challenges in large-scale cultivation and reliance on expensive animal-derived materials. To date, only one company (Pendulum) has produced a 100 M AFU (Active fluorescent units) live strain, but its efficacy and stability have not been fully validated.

Dietary strategies offer a more accessible and sustainable approach to enhancing its therapeutic potential. Certain foods have been shown to improve metabolic diseases, such as hyperlipidemia and diabetes, and increase the abundance of *A. muciniphila*, which may contribute to their beneficial effects. Metabolic disorders often disrupt the oral microbiome, resulting in dysbiosis that can worsen the severity of periodontal disease [39], are strongly influenced by diet, and proper nutrition that supports the growth of *A. muciniphila* can be an effective treatment strategy in reducing metabolic disease-induced periodontal disease.

Oats have a potential to provide beneficial effects in part due to their prebiotic influence on the gut microbiome. Oats nourish beneficial gut bacteria, potentially boosting levels of *A. muciniphila*. A study showed that ß-glucan from whole-grain oats has been found to mitigate HFD-induced hyperlipidemia by regulating bile acid metabolism [40] and modulate dyslipidemia by lowering total and LDL cholesterol levels [41]. Similarly, polyphenol-rich cranberry extract has improved obesity-related conditions in HFD-induced obese mice, reversing insulin resistance and hepatic steatosis while promoting the proliferation of *A. muciniphila* [42].

Moreover, polyphenols extracted from green tea [43] and omega-3 polyunsaturated fatty acids (EPA and DHA) from fish oil [44] have shown similar benefits, improving insulin resistance, reducing inflammation, and enhancing intestinal barrier function in HFD-induced obese mice. All these effects were associated with an increase of *A. muciniphila* which supports overall metabolic health. In addition, mulberry fruit’s polysaccharides have also demonstrated protective effects against diabetes such as limiting weight gain, lowering blood glucose, improving glucose tolerance, and increasing levels of *A. muciniphila* leading to alleviation of diabetes-associated symptoms [45].

Quinoa, rich in dietary protein, has been shown to decrease blood pressure in mice with HTN, with a shift in microbial structure towards that of normotensive mice and a higher abundance of *A. muciniphila* [46].

**Figure 5:**
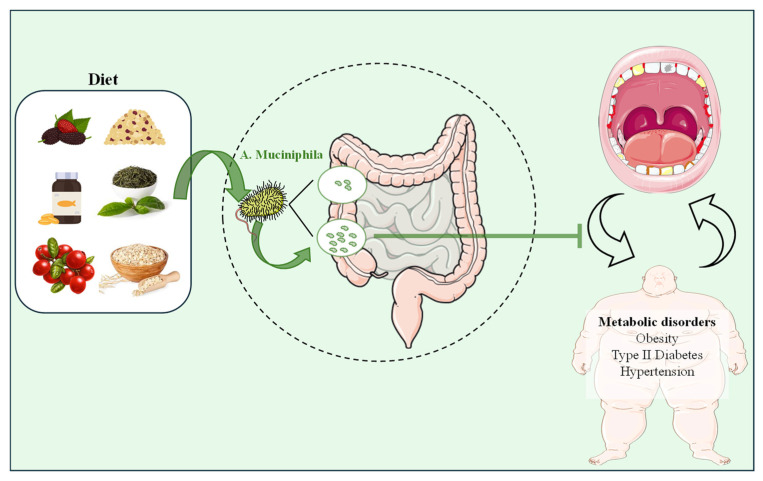
Impact of Specific Foods on Metabolic Diseases-induced periodontal diseases and *A. muciniphila* Abundance. Certain foods that help manage metabolic diseases like hyperlipidemia, diabetes, and hypertension also improve oral health. These foods increase the abundance of *Akkermansia muciniphila* (*A. muciniphila*), which may enhance their positive effects on systemic diseases and oral health. This suggests that dietary strategies could effectively address chronic conditions by leveraging *A. muciniphila*’s role in the gut microbiome to improve overall health.

The mechanism by which many dietary foods regulate metabolic disorders and increase the abundance of *A. muciniphila* largely stems from active components like polyphenols and polysaccharides [47]. These compounds are recognized as potential prebiotics, providing nourishment for *A. muciniphila*, and promoting its growth, enhancing its beneficial effects on metabolic health [47]. The beneficial effects of these foods on metabolic and oral health may stem from the increased abundance of *A. muciniphila* and its anti-inflammatory properties. By boosting *A. muciniphila* levels, these foods may help prevent and manage PD linked to metabolic disorders (Figure 5 and Table S2).

In conclusion, *A. muciniphila* presents significant potential as a therapeutic agent for addressing inflammation-related oral and systemic diseases. Its unique ability to modulate the microbiome and exert anti-inflammatory effects positions it as a promising candidate for innovative treatment approaches. The integration of *A. muciniphila* into clinical practice could revolutionize the management of PD and extend its benefits to a range of systemic conditions linked to chronic inflammation. By harnessing the therapeutic potential of *A. muciniphila*, we may be able to achieve substantial improvements in health outcomes, offering a targeted and holistic approach to managing both oral and systemic diseases through microbiome modulation. This could mark a significant advance in personalized medicine, emphasizing the critical role of the microbiome in maintaining overall health and preventing disease.

## 6. Study Limitations

The existing research on *Akkermansia muciniphila* (*A. muciniphila*) mainly involves controlled animal studies and small-scale human trials. The applicability of these findings to larger and more diverse populations is still uncertain, and there is a need for broader and more varied studies to confirm the generalizability of the results. Additionally, individual differences in microbiome composition can affect the effectiveness of *A. muciniphila* interventions, with variations in baseline microbiome profiles and dietary habits among participants potentially influencing outcomes. While the potential of *A. muciniphila* to modulate inflammation and improve oral health is promising, the exact mechanisms through which it operates are not yet fully understood. More research is required to elucidate how *A. muciniphila* interacts with other microbial species and host immune responses. Furthermore, the long-term effects and safety of *A. muciniphila* supplementation remain underexplored, with most studies concentrating on short-term outcomes. The practical challenges of cultivating *A. muciniphila* at a scale suitable for widespread clinical use need to be addressed, as current formulations, including those from specific companies, may face issues related to stability and efficacy. Additionally, the impact of dietary factors on *A. muciniphila* abundance and its effects on oral health is influenced by various elements such as nutrient availability and overall diet quality. Further investigation into the interactions between dietary components and *A. muciniphila* is necessary to refine dietary strategies. In both clinical and preclinical research, uncontrolled variables such as genetic differences, environmental factors, and concurrent use of other treatments could affect the results. Age also plays a significant role in regulating the levels of *A. muciniphila*. Researchers analyzed saliva samples from pediatric and adult populations using qPCR. They found that 29.8% of pediatric patients harbored AM, with a significantly higher prevalence in non-orthodontic children (42.3%) compared to orthodontic children (14.3%). In adults, the prevalence was lower, with non-orthodontic adults showing a higher presence of AM (21.3%) compared to orthodontic adults (12.8%). These findings suggest that both age and orthodontic treatment may influence the oral microbiome, with orthodontic brackets potentially reducing AM levels in both age groups. It is crucial to account for these variables to ensure the robustness and reliability of the findings (Table S3).

## Supplementary Materials

The following supporting information can be downloaded at: https://www.mdpi.com/article/10.3390/nu16183075/s1, Table S1: This table captures the multifaceted benefits of *Akkermansia muciniphila* across various aspects of health; Table S2: This table captures the key aspects of A. muciniphila’s clinical properties and its potential for therapeutic applications in both oral and systemic health; Table S3: References used in this paper with their key findings and outcome.
