# Supplementary material for: Akkermansia muciniphila as a Potential Guardian against Oral Health Diseases: A Narrative Review
Source: Nutrients. 2024 Sep 12;16(18):3075. doi: 10.3390/nu16183075 (PMC11434887; doi:10.3390/nu16183075)
Supplement: Supplementary file 1 [file nutrients-16-03075-s001.zip › nutrients-3194122-supplementary.pdf]

Property	Description	Benefits
**Anti-inflammatory Effects**	Reduces production of pro-inflammatory cytokines (e.g., TNF-α, IL-6) and increases anti-inflammatory cytokines (e.g., IL-10).	Mitigates inflammation in the gut and oral cavity; potentially reduces systemic inflammation.
**Regulation of Gut Barrier Integrity**	Enhances intestinal barrier function by breaking down mucins and producing SCFAs.	Maintains gut barrier integrity, preventing pathogen infiltration and reducing gut inflammation.
**SCFA Production**	Produces short-chain fatty acids (SCFAs) like acetate and propionate.	SCFAs exhibit anti-inflammatory properties and contribute to overall gut health.
**Oral Health Improvement**	Shown to reduce periodontal inflammation and bone loss in models of periodontal disease.	Helps manage periodontal diseases by reducing inflammation and tissue destruction.
**Impact on Metabolic Health**	Improves metabolic parameters including insulin sensitivity, cholesterol levels, and fat mass.	Potentially aids in managing obesity, type 2 diabetes, and related metabolic disorders.
**Effect on Systemic Diseases**	Can mitigate inflammation related to systemic conditions such as obesity, hypertension, and type 2 diabetes.	Offers therapeutic potential for various systemic diseases linked to inflammation.
**Dietary Influence**	Boosts abundance when supplemented with prebiotics from foods such as oats, polyphenol-rich cranberry extract, green tea, and quinoa.	Enhances overall health and potentially reduces disease risk through dietary interventions.
**Therapeutic Potential**	Potential for use in probiotic and prebiotic formulations for both oral and systemic health.	Provides a novel therapeutic avenue for managing chronic inflammation and related diseases.

Table S1: This table captures the multifaceted benefits of *Akkermansia muciniphila* across various aspects of health.

Clinical Property	Details
**Anti-Inflammatory Effects**	Downregulates pro-inflammatory cytokines and enhances anti-inflammatory responses, helping to mitigate tissue destruction and bone loss in periodontal disease (PD).
**Impact on Periodontal Disease (PD)**	Promising potential for managing PD by reducing inflammation and severity through modulation of the oral microbiota.
**Systemic Health Implications**	Improves insulin sensitivity, reduces cholesterol levels, and modulates inflammatory markers. May help manage obesity, type 2 diabetes (T2D), and hypertension (HTN).
**Probiotic and Prebiotic Formulations**	Integration in formulations could maintain balanced oral microbiota, reduce inflammation, and support overall health. Clinical trials needed for validation.
**Personalized Approaches**	Individual microbiome variability suggests that personalized treatments based on specific microbiota composition and inflammatory profiles could optimize outcomes.
**Mechanistic Understanding**	Further research is needed to understand interactions with other microbial species and host immune responses. Longitudinal studies are crucial for assessing long-term effects.
**Challenges in Cultivation**	Large-scale cultivation is challenging; currently, only one company (Pendulum) produces a live strain with limited validation of efficacy and stability.
**Dietary Strategies**	Foods such as oats, polyphenol-rich cranberry extract, green tea polyphenols, omega-3 fatty acids, and quinoa may enhance A. muciniphila levels and improve metabolic and oral health.
**Prebiotic Influence of Foods**	Certain dietary components like ß-glucan from oats and polyphenols from fruits promote A. muciniphila growth, which may help manage metabolic disorders and associated periodontal disease.

Table S2: This table captures the key aspects of A. muciniphila's clinical properties and its potential for therapeutic applications in both oral and systemic health.

Reference	Key Findings	Outcome
Deo, P.N.; Deshmukh, R. (2019)	Provides an overview of the oral microbiome and its fundamental aspects.	Outlines foundational knowledge on the oral microbiome.
Siddiqui, R. et al. (2023)	Highlights the increasing recognition of the oral microbiome's role in periodontal health and disease.	Emphasizes the importance of oral microbiota in periodontal conditions.
Lamont, R.J. et al. (2018)	Reviews dynamic interactions between the oral microbiota and the host.	Discusses how oral microbiota communities affect health and disease.
Moreno, C.M. et al. (2023)	Explores the immunomodulatory role of oral microbiota in inflammatory and allergic conditions.	Details the impact of oral microbiota on systemic inflammatory responses.
Sedghi, L. et al. (2021)	Examines key organisms and complex networks in the oral microbiome.	Focuses on the roles of specific microorganisms in oral health and disease.
Santacroce, L. et al. (2023)	Provides a comprehensive perspective on the oral microbiota in health and disease.	Summarizes current knowledge and perspectives on oral microbiota.
Kapila, Y.L. (2021)	Discusses the relationship between oral health and systemic conditions, focusing on special populations.	Highlights the connection between periodontal and systemic diseases.
Song, B. et al. (2023)	Akkermansia muciniphila inhibits periodontitis caused by Fusobacterium nucleatum.	Demonstrates the potential of Akkermansia muciniphila in preventing periodontal disease.
Raftar, S.K.A. et al. (2022)	Investigates the anti-inflammatory effects of Akkermansia muciniphila in liver injury models.	Shows therapeutic potential of Akkermansia muciniphila for inflammatory conditions.
Blaser, M.J. (2014)	Introduces the concept of the microbiome revolution.	Discusses the transformative impact of microbiome research.
Zhang, T. et al. (2019)	Reviews the potential of Akkermansia muciniphila as a probiotic.	Highlights the beneficial effects of Akkermansia muciniphila.

Reference	Key Findings	Outcome
Cani, P.D. et al. (2022)	Discusses Akkermansia muciniphila as a model for beneficial microorganisms.	Emphasizes the therapeutic potential of Akkermansia muciniphila.
Tan, J. et al. (2014)	Reviews the role of short-chain fatty acids in health and disease.	Details how short-chain fatty acids impact various health conditions.
Elzinga, J. et al. (2024)	Identifies the specific O-glycan binding of Akkermansia muciniphila to mucins.	Provides insights into the mechanisms of Akkermansia muciniphila's interactions.
Saraiva, M.; O'garra, A. (2010)	Reviews the regulation of IL-10 production by immune cells.	Explains mechanisms of IL-10 regulation and its implications.
Si, J. et al. (2022)	Reassesses Akkermansia muciniphila's role as a therapeutic bacterium.	Reaffirms the potential of Akkermansia muciniphila in clinical settings.
Bian, X. et al. (2019)	Shows that Akkermansia muciniphila ameliorates ulcerative colitis in mice.	Highlights therapeutic potential in inflammatory bowel disease.
Loesche, W.J. (1996)	Details the microbiology of dental decay and periodontal disease.	Provides a historical overview of dental microbiology.
Ruan, Q. et al. (2023)	Examines how Porphyromonas gingivalis affects atherosclerosis via immune pathways.	Investigates the link between oral bacteria and cardiovascular disease.
Olsen, I.; Singhrao, S.K. (2018)	Explores the heterogeneity of Porphyromonas gingivalis lipopolysaccharide lipid A in inflammation.	Analyzes variations in inflammatory responses due to bacterial lipids.
Xu, W. et al. (2020)	Reviews the roles of Porphyromonas gingivalis and its virulence factors in periodontitis.	Discusses the impact of specific bacterial factors on periodontal disease.
Shen, C. et al. (2023)	Compares oral prevalence of Akkermansia muciniphila in orthodontic vs. non-orthodontic patients.	Examines differences in Akkermansia muciniphila prevalence in different patient groups.

Reference	Key Findings	Outcome
Huck, O. et al. (2020)	Shows that Akkermansia muciniphila reduces inflammation and bone destruction caused by Porphyromonas gingivalis.	Demonstrates potential therapeutic effects of Akkermansia muciniphila in periodontal disease.
Mulhall, H. et al. (2022)	Reports that pasteurized Akkermansia muciniphila reduces inflammation induced by Porphyromonas gingivalis.	Supports the use of Akkermansia muciniphila in reducing systemic inflammation.
Arigbede, A.O. et al. (2012)	Reviews the connection between periodontitis and systemic diseases.	Summarizes evidence linking periodontal disease with systemic health conditions.
Malik, V.S. et al. (2013)	Reviews global obesity trends, risk factors, and policy implications.	Provides a comprehensive overview of obesity and related factors.
Abuqwider, J.N. et al. (2021)	Reviews the role of Akkermansia muciniphila in modulating obesity.	Highlights Akkermansia muciniphila’s impact on obesity and metabolic health.
Yang, M. et al. (2020)	Shows beneficial effects of newly isolated Akkermansia muciniphila strains on obesity and metabolic disorders.	Demonstrates positive effects of specific Akkermansia muciniphila strains on metabolic health.
Depommier, C. et al. (2019)	Provides proof-of-concept study on Akkermansia muciniphila supplementation in overweight and obese humans.	Highlights potential benefits of Akkermansia muciniphila in human obesity studies.
Rodrigues, V.F. et al. (2022)	Discusses Akkermansia muciniphila's role in attenuating inflammatory bowel disease, obesity, and diabetes.	Highlights the multifaceted benefits of Akkermansia muciniphila.
Schneeberger, M. et al. (2015)	Shows that Akkermansia muciniphila inversely correlates with inflammation and metabolic disorders in obesity.	Demonstrates the association between Akkermansia muciniphila and reduced inflammation in obesity.
Mills, K.T. et al. (2020)	Reviews global epidemiology of hypertension.	Provides an overview of hypertension trends and epidemiology.

Reference	Key Findings	Outcome
Yang, T. et al. (2015)	Links gut dysbiosis to hypertension.	Investigates how gut microbiota imbalances may affect blood pressure.
Al Khodor, S. et al. (2017)	Discusses the potential of microbiota in regulating blood pressure.	Explores the role of the microbiome in blood pressure regulation.
Chen, X. et al. (2019)	Reviews gut microbiota alterations and therapeutic interventions in heart failure.	Examines the impact of gut microbiota on heart failure and related therapies.
Ottman, N. et al. (2017)	Reviews the action and function of Akkermansia muciniphila in health and disease.	Provides insights into Akkermansia muciniphila's role in various health conditions.
Lakshmanan, A.P. et al. (2022)	Evaluates the impact of Akkermansia muciniphila on blood pressure regulation.	Discusses the evidence supporting Akkermansia muciniphila's effects on blood pressure.
Pirih, F.Q. et al. (2021)	Explores the association between metabolic syndrome and periodontitis, focusing on lipids, cytokines, and microbiome.	Highlights the interplay between metabolic syndrome and periodontal disease.
Duan, R. et al. (2021)	Investigates the effects of flavonoids from oat on hyperlipidemia via gut microbiota regulation.	Shows the impact of oat flavonoids on lipid metabolism and gut microbiota.
Xu, D. et al. (2021)	Compares prebiotic effects of oats and rice on blood lipids and gut microbiota.	Provides evidence of oats' prebiotic benefits compared to rice.
Anhê, F.F. et al. (2017)	Reports that cranberry extract reverses insulin resistance and hepatic steatosis independent of weight loss.	Demonstrates benefits of cranberry extract on metabolic health.
Jeong, H.W. et al. (2020)	Shows that green tea promotes growth of Akkermansia muciniphila.	Highlights the beneficial interaction between green tea and Akkermansia muciniphila.
Monk, J.M. et al. (2019)	Reports that fish oil supplementation improves intestinal health and systemic obesity phenotype.	Indicates benefits of fish oil on gut health and obesity.

Reference	Key Findings	Outcome
Chen, C. et al. (2018)	Examines how mulberry fruit polysaccharides modulate gut microbiota in obese diabetic mice.	Demonstrates the impact of mulberry polysaccharides on gut microbiota and metabolic health.
Guo, H. et al. (2021)	Reports quinoa protein's effect on blood pressure and fecal microbiota in hypertensive rats.	Highlights quinoa protein's potential in managing hypertension.
Xu, W. et al. (2024)	Reviews dietary nutrients and medicinal foods targeting Akkermansia muciniphila for metabolic disorders.	Discusses dietary strategies and their effects on Akkermansia muciniphila and metabolic health.

Table S3: references used in this paper with their key findings and outcome
